# Chih Tu: A pioneer of Xinjiang’s agricultural science

**DOI:** 10.1007/s13238-014-0096-2

**Published:** 2014-08-26

**Authors:** Fang Zhang

**Affiliations:** Institute for the History of Natural Sciences, Chinese Academy of Sciences, Beijing, 100190 China

Chih Tu was a pioneer of Xinjiang’s agricultural science and technology, the founder of Xinjiang’s agricultural education, and the first academician from Xinjiang to join the Chinese Academy of Sciences. In addition to being a distinguished plant pathologist and agriculturist, he was a communist fighter with a strong spirit and a revolutionary sentiment. Deeply influenced by revolutionary ideas and the Chinese Communist Party (CCP), he spent most of his life in the northwest part of China.

Chih Tu was born in a family of scholars in the city of Hwang-pih in Hu-Peh province on July 15, 1903.^1^ His grandfather Tao-Yung Tu was a scholar of late Qing Dynasty and a traditional private tutor who advocated new learning. His father Heng-Fu Tu was involved in business, but most of his uncles were scholars. Chih Tu himself was the uncle of Kuang-Chih Tu, a renowned geochemical scientist and expert on mineral deposits. Chih Tu received good education as a small boy. He attended Tsinghua School (the predecessor of Tsinghua University) in Beijing in 1915, which marked the start of his academic journey. During the period of the May Fourth Movement, like many other young patriots, he joined the march calling for democracy and went through the great movement of ideological liberation. His motivation to save the nation through science originated from his experiences during that time.

**Figure:**
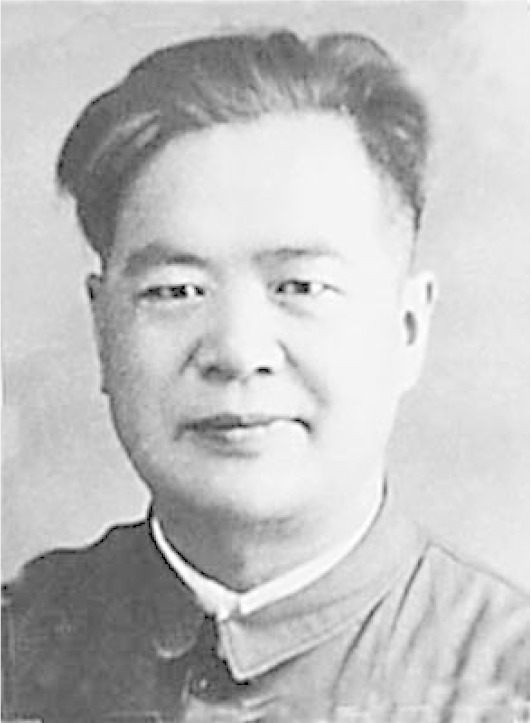
Chih Tu (1903–1976)

After graduating from Tsinghua, Chih Tu went to the United States for graduate study with his classmate Pei-Yuan Chou. With the ambition of revitalizing China’s agriculture by modern agricultural science, he planned to take agriculture as his major. He studied under the guidance of Professor Elvin Charles Stakman in the Department of Agriculture at the University of Minnesota. Stakman was a pioneering American plant pathologist and educator who established the methods for identifying and combating diseases of wheat and other important food crops. According to Dr. Stakeman, Chih Tu was one of his most distinguished students. He was extremely diligent and often spent entire nights conducting experiments. His memory, wisdom and talent were exceptional. Hsien-wen Li, a distinguished geneticist, described Chih Tu as an extraordinarily intelligent agriculturist. He earned a doctorate in 1929 for his dissertation titled “*Physiologic specialization in Fusarium Spp. Causing headblight of small grains*”.

With the ambition of saving his nation through science, Chih Tu returned to China in 1929. From 1929 to 1938, he worked at several universities and institutes. At Lingnan University, he devoted himself to scientific research and education in service to his country. He was appointed as a professor of plant pathology and put in charge of all agricultural work at the Agricultural Department of Henan University where many notable agriculturists worked. Chih Tu was the first to advocate use of large-scale agricultural machinery in Henan Province. In 1934, with an invitation from National Wuhan University, he returned to his hometown to help them establish a cotton experiment station at Wuhan University. In the following year, he took the position of the president of the Northwestern Agricultural School, which had recently been established at the time. It was during this period that he gradually realized that he should use practical techniques to save the nation. Accordingly, he established connections with Tien-yu Le, an active member of the CCP from whom he learned much about Marxism-Leninism, and began to serve for the CCP and participated in anti-Japanese propaganda activities.

With the invitation of Chung-yuan Tu, the Director of Xinjiang College, Chih Tu arrived in Dihua (now Urumchi) from Lanzhou in April, 1939. From then on, he devoted himself to Xinjiang’s agriculture and education for the rest of his life. At the beginning, he took the position of Provost at Xinjiang Senior Agricultural School, but later became the Director of the agricultural department at Xinjiang College and there after the Provost of Xinjiang College. He had high prestige among both teachers and students of all nationalities; the story of “Dr. Tu” was well known both at home and abroad. As a past Provost of Xinjiang College, Shen-hsien Kuo said, “I admire Chih Tu’s talent and ability. He is knowledgeable and versatile, honest and trustful, modest and unobtrusive. He says less but does more; he seems to be ordinary but actually brilliant.” He supported Marxism and the CCP and was imprisoned by the nationalist government for this reason. After his release, he was appointed as the Vice Director of Xinjiang College and continued to commit himself to revolutionary activities. His revolutionary zeal received much attention both within the CCP and across the country; as a result, he was invited to attend the First Chinese People’s Political Consultative Conference by President Mao met.

After the founding of New China, he became firmly committed to agricultural development and education. In 1952, Bayi Agricultural College (the predecessor of Xinjiang Agricultural University) was established, and Chih Tu was appointed as the Director. In order to find good faculty and staffs, he traveled around most of China in search of talented individuals. Some experts were so deeply impressed by him that they arrived in Xinjiang the day after they received his invitation. He championed the idea of linking theory with practice and combining education with production. In order to address the shortage of agricultural technology leaders, he took measures to facilitate interactions and communications between colleges and production groups. Furthermore, under his proposal, Xinjiang Institute of Agriculture, Forestry and Livestock, which later became Xinjiang Academy of Agricultural Science, was established in 1955 and he acted as Director.

Chih Tu was always pleased to support new things and to advocate for new technology. He proposed to establish the Utilization of Atomic Energy Research Institute and to use radiation for generating mutant plant varieties. He also advocated both crossbreeding and haploid breeding. To address poor water resources, he proposed using sprinkling irrigation and drip irrigation. Due to the shortage of frost-free period, he promoted plastic sheeting for rice seedling. Since Xinjiang has a small population but covers a large geographical area, he was determined to introduce mechanization into agricultural practices. In 1954, under his leadership, they had a bumper harvest of 20 thousand acres of cotton along the Manas River. This achievement not only set a new record but also dispelled the theory proposed by western scientists that cotton is not suitable for cultivation at northern latitudes. This achievement also expanded China’s cotton growing areas to the north. Furthermore, Chih Tu launched the journal *Agricultural Science in Xinjiang*. For his outstanding contribution to agriculture, he was elected to the Chinese Academy of Sciences in 1955.

He emphasized having a strong theoretical foundation and advocated free academic debate. During the 1950s, fierce controversy between Mendelian genetics and Lysenkoism caused some incidents in Xinjiang. At that time, some individuals who supported Lysenkoism destroyed the experimental plot and experimental instruments of researchers who advocated for Mendelian genetics. This rude, poor and terrible behavior was criticized by Chih Tu. He advocated for exchange of scientific results and civil academic debate. In particular, he invited several experts to Xinjiang to give lectures on Mendelian genetics. In the late 1950s, the current tendency of exaggeration also spread to Xinjiang, and some people claimed that per *mu* yield of wheat for an average year was ten thousands *jin.* Chih Tu denounced such unpractical behavior and conducted a comparative test to show that this was not the case.

Besides being an outstanding research scientist, he also knew several languages. Once, Xinjiang College had invited a group of agricultural and water conservancy experts from Russia to give lectures. However, no one was available for translation. Under this circumstance, Chih Tu took the responsibility of translation despite his busy schedule. After 1950, he took charge of translating foreign agricultural publications from English, French, German, and Russian into Chinese.

He also paid close attention to intellectual and career development of young individuals. He helped young teachers launch literature collection and research, collect specimen and build nursery gardens and herbariums. He was also very kind to his students and supported needy students for further studies. During the early 1930s, he supported the breeding experiment conducted by Pao-tsing Liu, who was a young teacher at Henan University, and supplied him with good varieties of wheat he had brought with him from America. This eventually helped Liu become successful in his breeding experiments. He educated many excellent science and technology students, some of whom later become leading scientists, including the wheat breeding expert Hung-chang Chao and the plant pathologist Ming-chi Wang.

In his later years, Chih Tu continued to work even though his health condition deteriorated towards the end of his life. He passed away on March 30, 1976. The news of his death was received with great sorrow by many who had been inspired by his enthusiasm and liberal ideas. He was buried in Urumchi Martyrs’ Cemetery. People are often seen standing in silence, showing respect to this “Prideman of Tianshan”. In order to honor the founder of the university and the pioneer of agricultural education in Xinjiang, a statue of Chih Tu was erected in the campus of Xinjiang Agricultural University.

**Figure:**
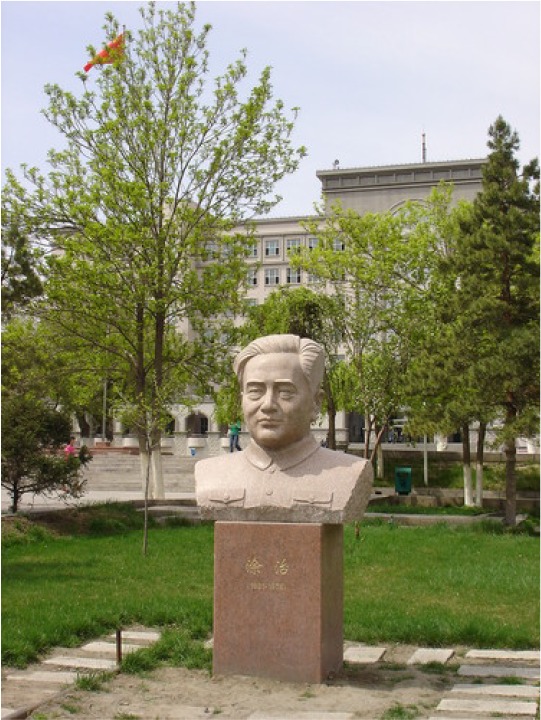
Chih Tu’s statue at Xinjiang Agricultural University

## FOOTNOTE

Relative Chinese names in this article: Chih Tu, 涂治; Tao-Yung Tu, 涂道泳; Heng-Fu Tu, 涂衡甫; Kuang-Chih Tu, 涂光炽; Pei-Yuan Chou, 周培源; Hsien-wen Li, 李先闻; Tien-yu Le, 乐天宇; Chung-yuan Tu, 杜重远; Pao-tsing Liu, 刘葆庆; Hung-chang Chao, 赵洪璋; Ming-chi Wang, 王鸣岐

## Footnotes

1 His date of birth is controversial. In a commemorative article written by his son and grandson, he was born in 1901; while in some other papers his birth date is August 20, 1903. However, in an archive from University of Minnesota, his birthday is July 15, 1903. Here follows this record.
